# P-804. Dulling Reflexes: Refining Criteria for Reflex Urine Cultures in Outpatient Areas and Emergency Departments to Decrease Inappropriate Antimicrobial Use

**DOI:** 10.1093/ofid/ofaf695.1013

**Published:** 2026-01-11

**Authors:** Andrea W Reed, Danielle Doughman, Kevin Alby, Ashley H Marx, Emily Sickbert-Bennett, Nikolaos Mavrogiorgos

**Affiliations:** UNCHealthcare, Bear Creek, North Carolina; University of North Carolina Medical Center, Chapel Hill, North Carolina; University of North Carolina, Chapel Hill, North Carolina; University of North Carolina Medical Center, Chapel Hill, North Carolina; UNC Medical Center, Chapel Hill, North Carolina; University of North Carolina Medical Center, Chapel Hill, North Carolina

## Abstract

**Background:**

Urinary tract infections (UTIs) are overdiagnosed and overtreated. Too-sensitive lab criteria for urine culture reflex contribute to the problem, leading to possible patient harm stemming from misdiagnoses and antibiotic overuse.

**Figure:**
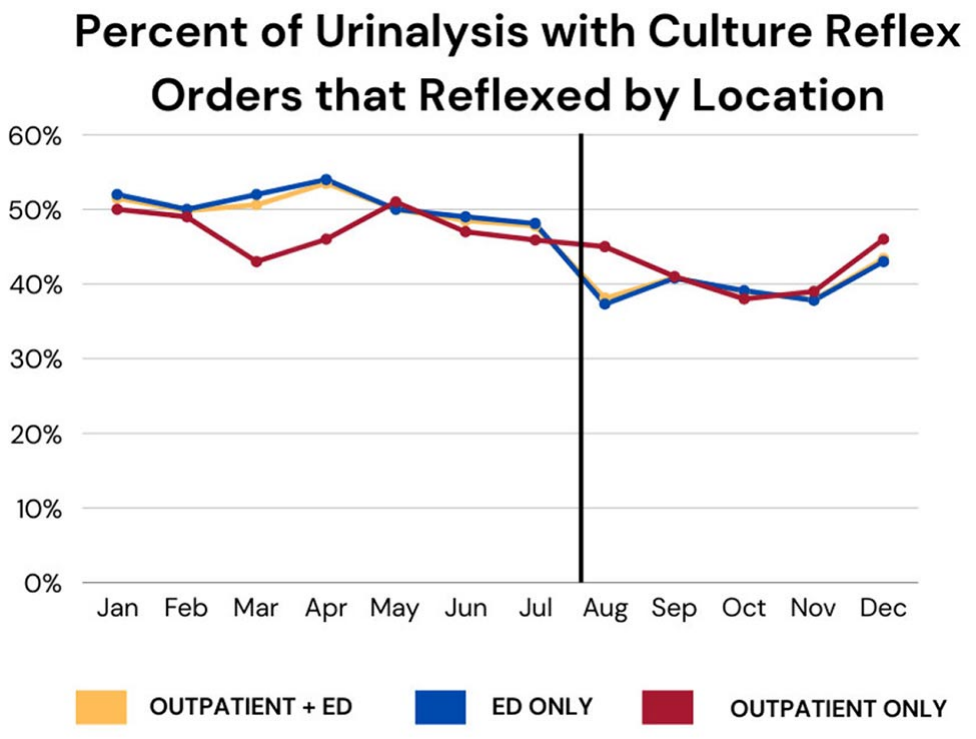
Percent of Urinalysis with Culture Reflex Orders that Reflexed by Location UAs that reflexed to culture fell from 51% (CI: 50-52%) pre-intervention to 40% [CI: 38-41%] post by refining culture reflex criteria.

**Methods:**

In July 2024, UNC Medical Center made urinalysis (UA) with culture reflex criteria changes in outpatient areas (OP) and the emergency department (ED): increase white blood cell counts (WBC) from 4 to 10/hpf; exclude positive bacteria; add new triggers for positive pregnancy test or absolute neutrophil count of < 0.5 x 10^9/L; lower patient age for exclusion from < 5 years to < 3 months; and keep leukocyte esterase or positive nitrites. Any single inclusion criterion in the UA triggered a reflex culture.

We conducted a retrospective pre- and post-study of patients with an outpatient- or ED-initiated urinalysis with culture reflex between 1/1-6/30/2024 (pre) and 8/1-12/31/2024 (post). EMR data was analyzed and descriptive statistics were calculated.

**Results:**

For all OP and ED, UAs that reflexed to culture fell from 51% [CI: 50-52%] in the pre-period to 40% [CI: 38-41%] post-period. In the ED, reflex cultures decreased from 51% [CI: 50-52%] to 40% [CI: 38-41%]. In OP, reflex culture decreases from 47.6% [44.7-50.6%] to 42% [CI: 38.9-45.5%] were not significant.

Changes averted 27% (1,472) of reflex culture orders that would have reflexed under the pre-period criteria. Of those, 91% were averted due to removal of the positive bacteria criterion, 21% due to WBC count, and 1% due to age.

For patients whose UAs did not reflex, 6% (279/5,372) were diagnosed with cystitis, UTI, or pyelonephritis within 28 days (51% were cystitis, 28% UTI, and 27% pyelonephritis). New urine culture orders were placed within 24 hours for 2% (119).

**Conclusion:**

Modifying reflex triggers for UA to reflex culture orders in ED decreased the number of inappropriate cultures performed.

**Disclosures:**

Kevin Alby, PhD, Gradientech: Grant/Research Support

